# VTE in ICU: Lessons from COVID-19

**DOI:** 10.1007/s44231-022-00016-1

**Published:** 2022-10-27

**Authors:** Xu Chu, Yimin Mao

**Affiliations:** 1 Department of Pulmonary and Critical Care Medicine, The First Affiliated Hospital of Henan University of Science & Technology, Jinghua Road 24, Luoyang, 471003 Henan Province China; 2 Luoyang Key Laboratory of Pulmonary Vascular Disease, The First Affiliated Hospital of Henan University of Science & Technology, Jinghua Road 24, Luoyang, 471003 Henan Province China

**Keywords:** VTE, ICU, Prevention, COVID-19

Since the establishment of the intensive care unit (ICU), it has been a place with a high incidence of thrombotic events. A study showed that venous thromboembolism (VTE) events were observed in 5.2% ICU patients, while the rate increased to 8.3% in COVID-19 patients in ICU [1]. The critically ill patients admitted to the ICU have specific features that need to be taken into account for the prevention and treatment of thrombosis. The three elements of thrombosis are vascular injury, hypercoagulability, and blood stasis. In the ICU, the three elements of thrombosis often manifest in a concentrated manner. The broken changes of the blood vessel often due to trauma, or intravenous operation. The changes in blood components include blood hypercoagulability that is secondary to the systemic inflammatory response syndrome, or coagulation system disorders. Hemorheological changes manifest as slow blood flow due to bed rest or sedation, or protective restraint. These changes often co-occur in ICU patients, ultimately leading to thrombotic events and making the ICU a high VTE risk area for patients.

Every patient admitted to the ICU has one or more of the following risk factors for VTE: advanced age, severe trauma, sepsis, APACHE II score > 12, surgery, long hospital stay before admission to the ICU, immobilization, mechanical ventilation, indwelling venous lines, blood purification treatment, use of muscle relaxants, sedatives and vasoconstrictors, and blood components supplement such as platelets. Of course, the concept of modern ICU has incorporated the idea of ​​"rapid recovery". It is required that patients should recover as soon as possible and keep their sitting position as much as possible, which could reduce the average length of stay in ICU, shorten the time that patients stay in the ICU, and accelerate patient turnover. The strong promotion of anticoagulation therapies has gradually reduced the risk of VTE in the ICU, however, there is still a remaining residual risk.

So, what are the risk factors for VTE in critically ill patients with COVID-19?

Since the beginning of 2020, the COVID-19 epidemic has spread around the world. Although most cases are mild, there are still many severe patients who need to be treated in the ICU. Research has shown that there are still more VTE events in these patients.

Patients with severe COVID-19 infection has experienced systemic inflammation. Neutrophils and other inflammatory cells are activated, inflammatory factors are produced at high levels, an inflammatory storm occurs, and the levels of C-reactive protein and procalcitonin increase. Both increased C-reactive protein and procalcitonin are risk factors for VTE. Subsequently, there are sepsis-related coagulation disorders. This indicates that the coagulation system is abnormal, hyperfibrinolysis occurs, and D-dimer is significantly increased [2]. Peak value of D-dimer is also an important risk factor for VTE. Indicators of disease severity, length of hospital stay, mechanical ventilation, use of inotrope and muscle relaxants, SOFA score, and use of ECMO were also risk factors for VTE [2]. In addition, underlying diseases such as chronic obstructive pulmonary disease, asthma, structural heart disease, and chronic kidney disease are also high-risk factors for VTE [3]. Patients with severe pneumonia caused by COVID-19 have a similar risk of developing VTE as other ICU patients. This finding is not difficult to understand as severe cases of COVID-19 essentially still have severe pneumonia. Therefore, they are no different from other ICU patients who are not with COVID-19. A major feature of ICU patients is that the severe pneumonia, sepsis, or shock that occurs in these patients is secondary to any disease and has a certain degree of similarity in pathophysiology or pathogenesis.

To prevent the occurrence of VTE, the risk of VTE needs to be assessed. In clinical practice, we have noticed that doctors in not only ICU, but also many departments (e.g., oncology, obstetrics, gynecology, respiratory department, and orthopedics) are aware of VTE prevention and control with patients at high risk of VTE. The awareness has increased significantly compared with previous years, and even low-risk departments have a strong awareness of VTE prevention. For example, in our hospital, all adult patients need to undergo VTE assessment after admission. Based on the risk, the physicians will prescribe corresponding preventive measures, and then enter the medical history collection or other doctor's prescriptions in the patient’s record. The national program for prevention and management of pulmonary embolism and deep venous thrombosis, led by the Chinese-Japan Friendship Hospital, aims to promote a new program of preventing VTE events across China. Our hospital is one of the first of excellent center units in this program.

So, how to prevent and manage VTE in severe COVID-19 patients?

An expert consensus on preventive management measures for VTE risk in admitted or critically ill COVID-19 patients has just been published in the Chest journal [4]. For patients with COVID-19 requiring hospitalization, the use of therapeutic doses of heparin, including unfractionated heparin and low molecular weight heparin (LMWH), rather than standard doses of heparin, is recommended for prophylactic anticoagulation. Because of the higher dose of heparin used, the balance of risk and benefit with bleeding and prevention of thrombosis needs to be weighed [5]. This recommendation is mainly based on the HEP-COVID Randomized Clinical Trial [6], which was originally designed to examine the optimal dose of thromboprophylaxis in high-risk patients with VTE. The trial evaluated the efficacy and safety of thromboprophylaxis in COVID-19 patients of therapeutic doses of heparin compared with standard doses of heparin. The primary efficacy outcome of this study was venous thromboembolism, arterial thromboembolism (ATE), or all-cause death, and the primary safety outcome was the presence of major bleeding at 30 days. In this randomized clinical trial, therapeutic doses of LMWH reduced major thromboembolism and death compared with institutional standard heparin thromboprophylaxis in hospitalized patients with COVID-19 with high elevated D-dimer levels. No difference was observed in ICU patients [6]. However, in the expert consensus published in the Chest journal, a standard dose of heparin is also recommended in severe COVID-19 patients for the prevention of VTE, although the level of evidence for this recommendation is relatively low.

At present, the global COVID-19 epidemic is still ongoing. Many mutant strains appear all over the world, such as the omicron mutant strain. However, the number of COVID-19 cases has decreased significantly, due to the gradual reduction of the virulence of the virus. In addition, the effect of herd immunity gradually shows due to the widespread vaccination of the population [7].

The basic mechanism of severe COVID-19 is still a kind of severe pneumonia, and the ICU is the main battlefield for medical staff to treat severe pneumonia. Facing the complex situation of VTE prevention and treatment in the ICU and dealing with new challenges brought by COVID-19, clinical trials on VTE prevention and treatment in severe COVID-19 patients are in progress. We look forward to more data providing more evidence to support the decision of specific anticoagulant doses and therapeutic doses in clinical practice.

## Data Availability

Not applicable.
